# Effectiveness of high-frequency cervical spinal cord stimulation in the treatment of refractory trigeminal neuropathy: A case report

**DOI:** 10.1097/MD.0000000000022304

**Published:** 2020-10-02

**Authors:** Daniela Floridia, Francesco Cerra, Francesco Corallo, Marcella Di Cara, Salvatore Spartà, Giovanni Nania, Alessia Bramanti, Placido Bramanti, Antonino Naro

**Affiliations:** Istituto di Ricovero e Cura a Carattere Scientifico (IRCCS) Centro Neurolesi Bonino-Pulejo, Messina, Italy.

**Keywords:** cervicomedullary junction, debilitating pain, quality of life, spinal cord stimulation, trigeminal nerve neuropathy

## Abstract

**Rationale:**

Treatment of chronic neuropathic pain in the head and face regions presents a challenge for pain specialists due to the lack of reliable medical and surgical approaches.

**Patient concerns:**

A 62-year-old patient came to our attention for an intense facial pain secondary to a lesion of the right trigeminal nerve (all branches) due to a petroclival meningioma.

**Diagnoses:**

The patient also presented with gait impairment as well as a deficit of the right facial, auditory, trochlear and abducens cranial nerves.

**Interventions:**

Conventional medical management (CMM) as well as tonic SCS were already adopted but they all dramatically failed. We intervened with the use of high-frequency (10 kHz) spinal cord stimulation (HFSCS) at the cervicomedullary junction (CMJ). The patient was thus provided with HFSCS at the CMJ. Pain and quality of life (QoL) were assessed 1 and 3 months after implantation. We also tested the trigeminal-facial reflex responses.

**Outcomes:**

HFSCS led to a full relief from the debilitating electric shocks like pain in the right hemiface, even though a background dull pain appeared. The gradual addition of pregabalin helped in fully relieving the painful symptomatology, with a significant improvement in QoL. Moreover, sensitivity amelioration on the inner portion of the mouth allowed the patient to start feeding again also using that side of the mouth. These findings were paralleled by a significant reshape of trigeminal-facial reflex responses suggesting an inhibition of nociceptive sensory inputs at brainstem level following HFSCS.

**Lessons:**

This is the first report suggesting the usefulness of HFSCS at the CMJ in neuropathic pain due to trigeminal nerve neuropathy non-responsive to tonic SCS and CMM.

## 1 Introduction

Chronic pain is a debilitating condition for millions of people worldwide. Such form of pain can be due to different medical conditions, including peripheral nerve disorders (such as complex regional pain syndrome) and primary pain disorders (e.g., neuropathic pain and fibromyalgia).

Both medical and surgical treatment strategies have been used in the treatment chronic pain. However, a non-negligible percentage of patients with chronic pain deserves second-level approaches, including spinal cord stimulation (SCS).^[1]^ This consists of stimulating the spinal cord at segmental or cervicomedullary junction (CMJ) level by means of electric impulses that have a neuromodulatory effect on the afferent pathways. Indications for cervical SCS include brachial plexus lesions, complex regional pain syndrome, degenerative disc disease, failed neck surgery syndrome, chronic radiculopathy, and post-herpetic neuralgia (PHN); those for SCS at the CMJ include trigeminal deafferentation pain, trigeminal neuropathic pain, and occipital neuralgia. About that, the spinal trigeminal tract and nucleus (particularly the caudal spinal nucleus, SpC) form the anatomic basis for CMJ SCS. In fact, these structures contain the cell bodies of second-order neurons that vehicle pain and temperature input from the ipsilateral face. More in detail, the sensory neurons of the trigeminal ganglion (TG) innervate the head, face and its adjacent sentient structures such as teeth, sinuses, dura mater, cornea, and temporo-mandibular joint (TMJ). TG sensory neurons are most relevant for orofacial pain and headaches, forms of pain that share a clinical hallmark of significant mechanical allodynia (e.g, in trigeminal neuralgia, atypical face pain, dental pulpits, keratitis, TMJ disorder, trigeminal PHN, and migraine).[^2^,^3^]

Painful orofacial cues are detected by nociceptive TG neurons and relayed to the SpC. As for the spinal cord dorsal horn, peptidergic, and non-peptidergic C-fibers of TG nociceptive neurons project to the superficial layers of SpC including lamina I and lamina II,^[4]^ whereas TG low-threshold mechanoreceptor (LTMR) neurons project to deeper laminae III-V in SpC.

However, the role of SCS at the CMJ for facial pain remain largely uncharted.^[5]^ Further, the neurophysiological basis is still unclear.

Herein, we report on the use of high-frequency (10 kHz) SCS (HFSCS) at the CMJ in a patient suffering from right cranial trigeminal nerve neuropathy after removal of a petroclival meningioma. The patient achieved a full relief from debilitating pain in his hemiface. These findings were paralleled by a significant reshape of trigeminal-facial reflex responses suggesting an inhibition of nociceptive sensory inputs at brainstem level following HFSCS.

## 2 Case report

**Figure 1:**
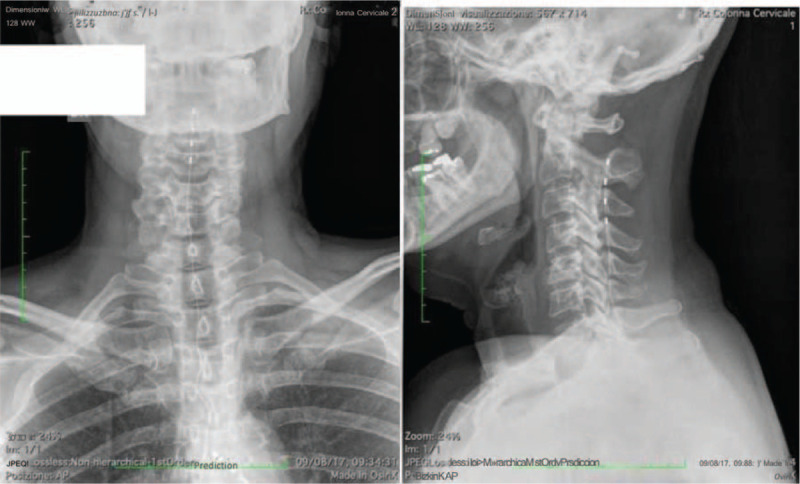
Blink reflex of the trigeminal nerve.

**Figure 2:**
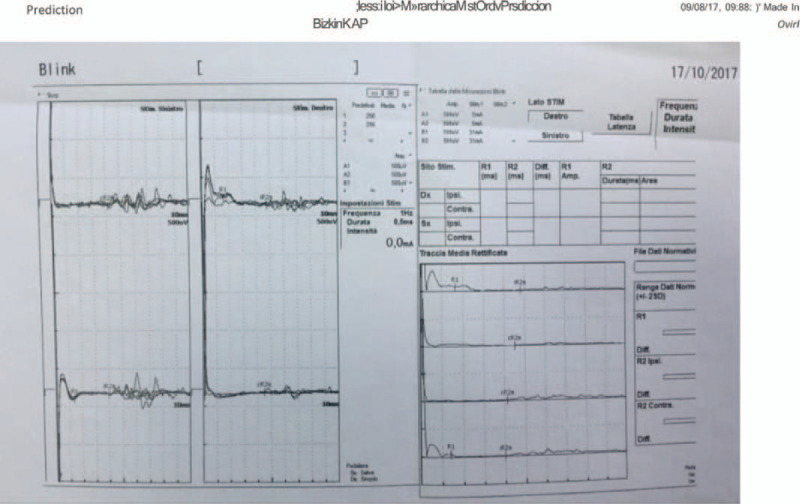
Cervical (C1-C2) lead position with tonic SCS.

The patient is a 62-year-old woman who began in 2004 to complain of an intense pain in correspondence of the II and III branch of the right trigeminal nerve, upper and lower right hemi-lip numbness, and a burning sensation in the right half of the mouth (Table 1). Pain onset was abrupt and showed an aggravation trend. Thus, she performed a brain Computerized Tomography (CT) scan that was normal. According to the clinical and neurological examination, the patient was diagnosed with trigeminal neuralgia and CMM therapy with carbamazepine (200 mg 3 times/day) and gabapentin (300 mg 3 times/day) was initiated. Nonetheless, the patient reported no symptoms remission. She then performed dental consultancy and was subjected to extraction of the last 2 molars of both right dental arches. With time, the patient began to complain of hypoesthesia of the II and III branches of the right trigeminal nerve, dysphagia for both solids and liquids, episodes of blurred bilateral vision lasting a few seconds with orthostatic hypotension. On September 2006, she performed a contrast-enhanced brain Magnetic Resonance Imaging (MRI) that showed the presence of an extra-axial expansive lesion likely to be a petroclival meningioma. On November 2006, she underwent surgery to remove the lesion in an Institute in Milan. Neurological examination following surgery disclosed a deficiency of the right abducens and trochlear nerves, right hearing loss, mild dysarthria and dysphagia; moreover, herpes labialis and oral candidiasis were reported. At the 1-month follow-up, a partial deficiency of the right facial nerve and keratoconjunctivitis in the right eye were also appreciable. Moreover, the patient reported burning dysesthesia localized to all right trigeminal branches and typical right trigeminal neuralgia in the second branch. CMM therapy with amitriptyline (30 mg/day) was started, in addition to carbamazepine (at the same dosage practiced before) and analgesics whenever needed (not specified). Nine months later (September 2007), the patient underwent thermorhizotomy of the interested branches with a slight improvement of the symptomatology. An intrathecal morphine pump was implanted on January 2008, but the patient experienced serious reactions to morphine that convinced to a switch to ziconotide on March 2008. However, the patient was sent back to a pain therapist because she reported nausea and vomiting, thus preventing ziconotide dosage increase. In the same year, the patient underwent radiosurgery (Gamma Knife or Cyber Knife) with no benefits. The patient continued the same CMM up to 2013, when a SCS with tonic stimulation was implanted at C3-C4 level. The patient reported paresthesia on the right side with a 30% reduction in pain intensity. This was however followed by no further improvements. Moreover, paresthesia became bothersome with time, spreading also to the lower limbs, thus inducing clinicians to turn the device off. In May 2017, the patient came to our attention. Neurological examination disclosed neuropathy and neuralgia of all branches of the right trigeminal nerve, and an impairment of the right trochlear, abducens, facial and auditory nerves. CMM was nearly ineffective to control pain and right-side hypoesthesia. The numeric rating scale (NRS; 0 = no pain; 10 = the worst pain imaginable) was 10. In particular, the patient described her pain as debilitating and characterized by electric shocks and allodynia on the II and III branches. She cannot feed herself adequately due to difficulty in chewing or speaking lasting since long time. In addition, the patient was experiencing sleep disorders and started making regular use of hypnotic-sedatives. A brain MRI on August 2017 confirmed the presence of residual known pathological tissue following the ablation of the right petroclival meningioma, which was morpho-volumetrically substantially unchanged compared to the previous control on December 2011. A blink reflex (BR) examination disclosed delayed ipsilateral and contralateral responses when stimulating the right supra-orbital nerve, thus confirming the dysfunction of the right trigeminal nerve (Fig. 1). CMM therapy included pregabalin (75 mg bid), pantoprazole (20 mg/day), alprazolam (0.25 mg bid), lacosamide 50 mg (1 cp bid), amitriptyline (35 mg/day). On September 2017, we re-positioned the SCS leads from C3-C4 (Fig. 2) to C1-C2; tonic stimulation was maintained. However, no improvements were reported at the end of the trial period, while paresthesia became bothersome and spread to the lower limbs.

**Table 1:**
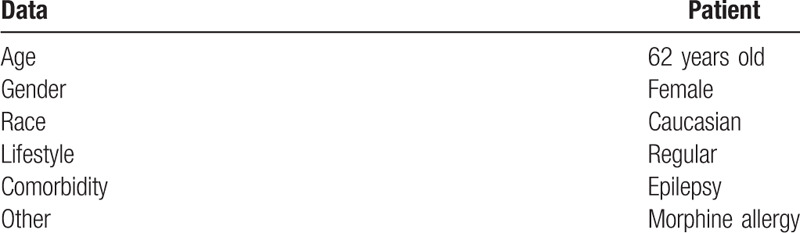
Patient demographic data and specific information.

**Figure 3:**
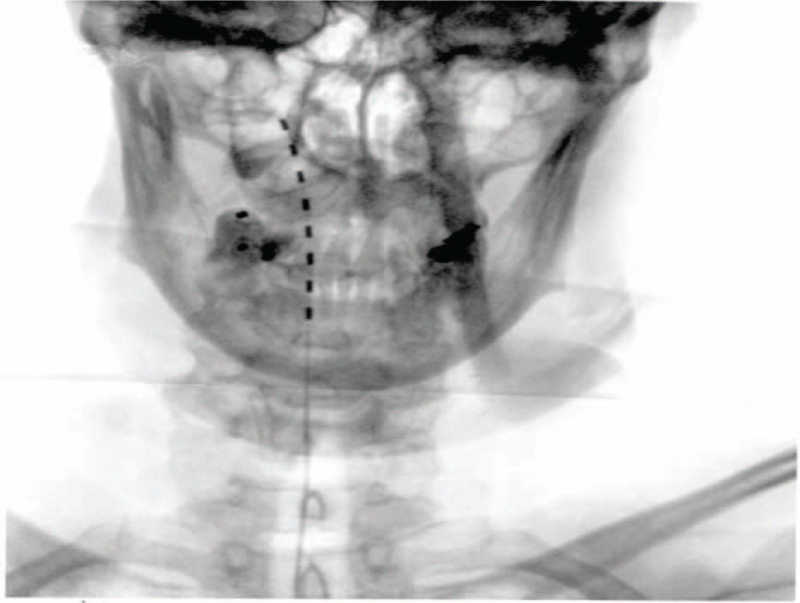
Cervical (C1-C2) lead position with HFSCS.

On December 2018, we provided the patient with an HFSCS trial (Fig. 3). Prior to implantation, routine blood tests and cardiological examination with ECG were required. Furthermore, an X-ray of the cervical spine was performed to check the post-intervention electro catheters positioning. Soon after HFSCS implantation, the patient had an improvement in the electric shocks like pain component on the inner side of right cheek up to a complete pain relief within 15 days, so that she could again brush her right dental arch and chew without feeling the strongly debilitating shocks. On January 2019, the patient underwent a permanent HFSCS implant.

## 3 Results

The HFSCS completely eliminated the electric shocks like pain component, but it also unmasked a background dull pain during the 3 months of follow up. This type of pain was treated with the addition of pregabalin (250 mg/day) to HFSCS and CMM. This combination led to a complete pain relief (Table 2). The quality and duration of sleep has also improved. Evaluation of compliance and tolerability of the intervention, including adverse events was evaluated and the patient showed good compliance and tolerability to surgery. Clinical improvements were still appreciable at the 3-month follow-up after HFSCS permanent implant (Tables 3 and 4). The QoL questionnaire showed a significant improvement especially in general pain, mental health, and emotional limitation role. Furthermore, there was a reduction in anxiety-depressive symptoms and an improvement in patients mood.

**Table 2:**
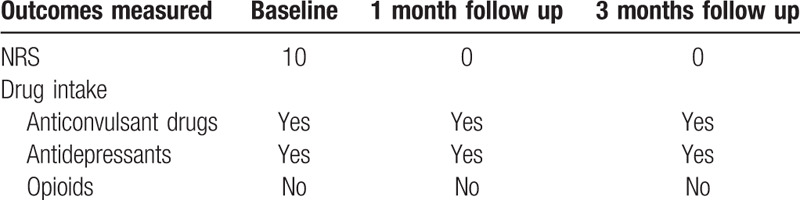
Outcomes measured at baseline, 1 and 3 months after HFSCS.

**Table 3:**
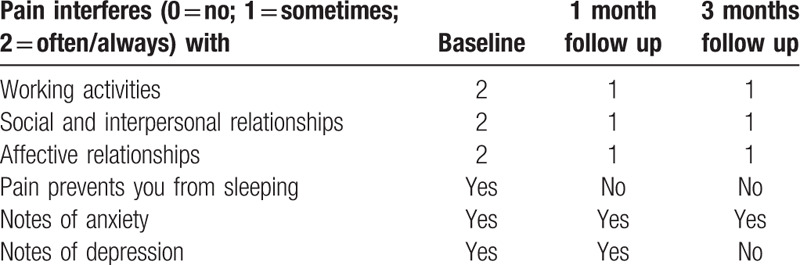
Neuropsychological test results (MIDI) at baseline, 1 and 3 months after HFSCS.

**Table 4:**
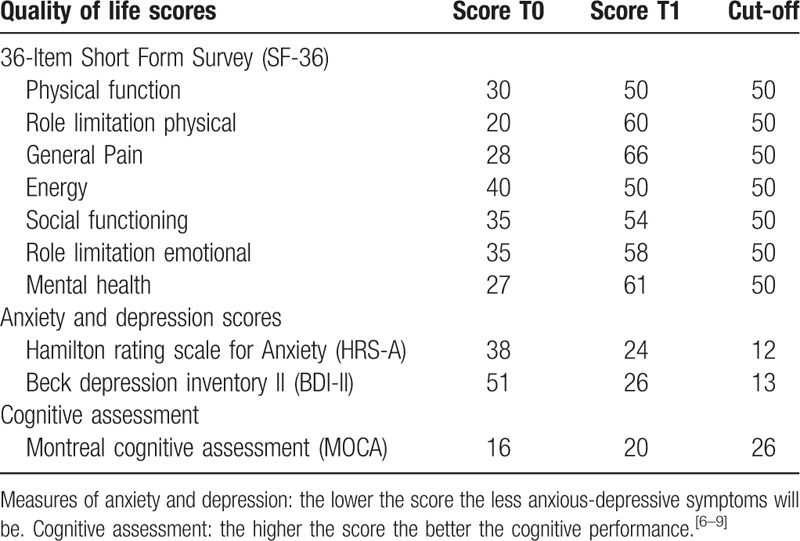
36-Item Short Form Survey (SF-36): the higher the score the better the QoL.

Routine BR (mediated by non-nociceptive myelinated Aß fibers), was normal at the follow-up visit. We therefore performed a nociceptive BR (nBR), which is mediated by nociceptive Aδ-and C fiber, given that several studies indicate that nBR could be helpful to assess pain in trigeminal neuralgia. nBR was investigated following nociception specific electrical stimulation on both sides of the face and in affected division of the trigeminal nerve with the SCS device first switched off (from at least 2hours) and then turned on (testing was performed 1 hour later). Two Ag-AgCl disk electrodes were fixed over the supraorbital nerve of the left and right side (in distinct sessions, approximately 2 cm apart each other). We first set the pain threshold by ascending and descending the stimulation intensity of 0.2 mA steps. The stimulation intensity to evoke nBRs was set at twice the individual pain threshold. We delivered 15 stimuli per side, made of triple pulse, monopolar square wave, duration 0.5 milliseconds (ms), pulse interval 5 ms; the interstimulus interval was set to 12 to 18 seconds (sec), pseudo-randomized in 1 session, and at less than 5 seconds, so to induce habituation response. These were recorded using Ag-AgCl disk electrodes fixed over the infraorbital surface, referenced to the orbital rim. Signal were sampled at 2.5 kHz, bandpass filtered at 1 Hz to 1 kHz, with a sweep-length of 300 ms (CED1401plus, Signal, Cambridge Electronic Design, UK). Once the 15 sweeps were averaged, we set nBR onset latencies and peak-to-peak amplitude. At rest, both nBR onset latencies and peak-to-peak amplitude were normal, whereas response habituation was missing (amplitude modulation induced by repetition = 1). One hour after having switched “ON” the SCS device, we found a reduction of 27% of the peak-to-peak amplitude of nBR and a restoration of response habituation (amplitude modulation induced by repetition = 0.4).

## 4 Discussion

Neuromodulation is a valid alternative approach to treat refractory pain when pharmacological and surgical treatments have failed. Further, CMM therapies are not effective in some cases and can cause substantial side effects. In literature, there are works on the use of SCS in the treatment of trigeminal neuralgia that report a pain relief between 50% and 70%.^[10]^

Our clinical case suggests the effectiveness of HFSCS at the CMJ with the addition of pregabalin in a patient with neuropathy and neuralgia of the right trigeminal nerve after 3 months of HFSCS permanent implant.

The placement of the octopolar electro-catheter in the peridural space at C1-C2, using a Thuoy needle, connected to subcutaneously implantable pulse generator (IPG) with stimulation at 10 kHz, induced a full relief of the paroxysmal pain in the right side with no improvement in the underlying dull pain, that was clearly unmasked during trial follow up and perceived by the patient once the main paroxysmal 1 ameliorated. This was however fully controlled by the administration of pregabalin in addition to HFSCS.

The anatomic target of HFSCS may be the deepest portion of the posterior horns of the spinal cord (deep dorsal horn). HFSCS are supposed to reduce the activity of wide dynamic range neurons and of excitatory neurons, while increasing the activity of inhibitory interneurons. This seems to occur at a greater extent than with other types of SCS.

In this case, the HFSCS could act in 2 ways:

- Through an action on LTMR (low threshold mechanosensory neurons) neurons placed on the deep layer of the SpC in lamina III and IV, and this could be responsible for the absence of paresthesia given that the LTMRs receive Aβ fiber inputs.
- Through targeting the non-peptidergic nociceptive neurons in the TG and the peptidergic C-fibers in the superficial layer of the SpC in lamina I and II.

What may be the final target of stimulation is the spinal tract of the trigeminal nerve formed by the large diameter fibers (Aβ) conveying discriminative tactile sensitivity (entering the pontine nucleus of the trigeminal nerve), the medium diameter fibers (Aδ) and many thin unmyelinated fibers (C) of the sensory root which always descend caudally from the pontine nucleus and convey the superficial pain, thermal and tactile sensitivity. Indeed, the pars caudalis is in the lower end of the medulla oblongata and in the first 3 cervical myelomers of the spinal cord.

The BR data mediated by non-nociceptive myelinated Aβ fibers, support this hypothesis. Indeed, the lack of amplitude modulation of the Aβ responses is in line with the involvement of the pars SpC, which is supposed to mediate nBR: HFSCS at CMJ modified selectively and unilaterally the nBR evoked by stimulating the V1 branch. This also suggests that HFSCS at the CMJ may exert a clinically relevant effect by decreasing Aδ-C input. This is analogous to the suppression of cortical or subcortical somatosensory evoked potential (SSEP) responses induced by SCS when placing the Thuoy electrode laterally at cervical level.

## 5 Conclusion

This is the first report suggesting the usefulness of HFSCS at the CMJ in neuropathic pain due to trigeminal nerve neuropathy non-responsive to tonic SCS and CMM. Even though randomized controlled trials are required to fully assess the indications and outcomes of HFSCS at the CMJ and to compare it with other therapeutic approaches, there is promising evidence to consider our approach as an effective treatment for patients with trigeminal neuropathic pain. Moreover, it has been recently published a cost-effectiveness analysis on HFSCS in failed back surgery syndrome (FBSS) that demonstrated using the Markov model that HFSCS is not only an effective treatment but it is also a cost-effective choice providing greater number of quality adjusted life years (QALYs) compared to other interventions.^[11]^ Indeed these latter data should promote together with further clinical evidences on HFSCS for different unmet or partially unmet conditions new decision trees that would promote patients well-being as well as resource allocation.

## Author contributions

**Conceptualization:** Daniela Floridia.

**Data curation:** Francesco Cerra.

**Formal analysis:** Antonino Naro.

**Investigation:** Francesco Corallo, Marcella Di Cara.

**Methodology:** Alessia Bramanti.

**Supervision:** Salvatore Spartà.

**Validation:** Placido Bramanti.

**Visualization:** Placido Braamnti, Daniela Floridia.

**Writing – original draft:** Marcella Di Cara.

**Writing – review & editing:** Daniela Floridia, Francesco Corallo.
